# Age-Related Reference Intervals for Blood Amino Acids in Thai Pediatric Population Measured by Liquid Chromatography Tandem Mass Spectrometry

**DOI:** 10.1155/2018/5124035

**Published:** 2018-05-06

**Authors:** Jaraspong Uaariyapanichkul, Sirinuch Chomtho, Kanya Suphapeetiporn, Vorasuk Shotelersuk, Santi Punnahitananda, Pannee Chinjarernpan, Orapa Suteerojntrakool

**Affiliations:** ^1^Division of Nutrition, Department of Pediatrics, King Chulalongkorn Memorial Hospital, The Thai Red Cross Society, Bangkok 10330, Thailand; ^2^Pediatric Nutrition STAR (Special Task Force for Activating Research), Department of Pediatrics, Faculty of Medicine, Chulalongkorn University, Bangkok 10330, Thailand; ^3^Center of Excellence for Medical Genetics, Department of Pediatrics, Faculty of Medicine, Chulalongkorn University, Bangkok 10330, Thailand; ^4^Excellence Center for Medical Genetics, King Chulalongkorn Memorial Hospital, The Thai Red Cross Society, Bangkok 10330, Thailand; ^5^Division of Neonatology, Department of Pediatrics, Faculty of Medicine, Chulalongkorn University, Bangkok 10330, Thailand; ^6^Center for Medical Diagnostic Laboratories, Faculty of Medicine, Chulalongkorn University, Bangkok 10330, Thailand; ^7^Division of Ambulatory, Department of Pediatrics, King Chulalongkorn Memorial Hospital, The Thai Red Cross Society, Bangkok 10330, Thailand

## Abstract

**Background:**

Age, race, and analytic method influence levels of blood amino acids, of which reference intervals are required for the diagnosis and management of inherited metabolic disorders.

**Objectives:**

To establish age-specific reference intervals for blood amino acids in Thai pediatric population measured by liquid chromatography tandem mass spectrometry (LC-MS/MS).

**Methods:**

A cross-sectional study of 277 healthy children from birth to 12 years was conducted. Anthropometric, clinical, and dietary information were recorded. Dried blood spots on a filtered paper were used for measurement by derivatized LC-MS/MS. Factors that might affect amino acids such as fasting time and dietary intake were analyzed using quantile regression analysis.

**Results:**

Levels of thirteen blood amino acids were reported as median and interval from 2.5th–97.5th percentiles. Compared with those of Caucasian, most blood amino acid levels of Thai children were higher. Compared with a previous study using HPLC in Thai children, many amino acid levels are different. Glycine, alanine, leucine/isoleucine, and glutamic acid sharply decreased after birth. Citrulline, arginine, and methionine stayed low from birth throughout childhood, whereas phenylalanine was at middle level and slightly increased during preadolescence.

**Conclusion:**

Reference intervals of age-specific blood amino acids using LC-MS/MS were established in the Thai pediatric population. They diverge from previous studies, substantiating the recommendation that, for the optimal clinical practice, age-specific reference intervals of amino acids should be designated for the particular population and analysis method.

## 1. Introduction

Amino acids are the basic structural units of protein. The diversity of amino acids provides variability in the function and structure of proteins. Some amino acids cannot be synthesized by the body. Therefore, they are considered essential constituents of the diet for maintenance of health and growth [1].

Inborn errors of metabolism (IEM) are a complex and heterogeneous group of genetic disorders, caused by a defect in a metabolic pathway, leading to malfunctioning metabolism and/or the accumulation of toxic intermediate metabolites. IEM can be presented at any age with nonspecific clinical manifestations, complicating diagnostic pathways. Furthermore, consequences are severe, causing morbidity and mortality in clinical practice, especially in pediatrics [2]. Although each disorder is individually rare, their cumulative incidence is substantial; an incidence of 1 in 2500–5000 live births has been shown to be upwards of 1 in 800 [2, 3]. In Thailand, from a retrospective study, the estimated pediatric patients with clinically suspected IEM are approximately 2–4%. However, in high-risk infants and children, the prevalence rates of 11.2% were reported [4–6]. Nevertheless, no study has been carried out to identify the magnitude of the problem in a systematic way [7]. As the delay in the diagnosis can result in irreversible outcomes [8], measurement of the levels of amino acids in the plasma is useful in the early diagnosis for the disorders to be treated promptly and for continuous monitoring of patients. Nutritional management includes limiting the substrates which trigger the symptoms while preventing their deficiencies. Patients will be assigned the type and amount of diets that can be taken without causing harm while maintaining adequate growth under the supervision of a nutrition specialist [9].

Analysis of amino acids is considered to have an extremely important role in the diagnosis and care of patients, detecting the type and amount of amino acids that are abnormal to identify the inborn errors of metabolisms, called aminoacidopathies. Originally, ion-exchange chromatography coupled with postcolumn ninhydrin or liquid chromatography combined with postcolumn derivatization using ninhydrin and UV detection was used [10] but suffered from a long analysis time, instability of reagents, and cross-reactivity with other constituents of the biological sample. Currently, there are several analytical methods, such as high-performance liquid chromatography (HPLC), gas chromatography (GC), and mass spectrometry (MS) [11]. Some laboratories in Thailand can perform HPLC, but there may be some drawbacks such as limited types of amino acids, long separation time, and high interlaboratory variation [12].

The use of two mass spectrometers in tandem (MS/MS) enables control of the formation of molecular and fragment ions. The combination of molecular mass (or mass-to-charge ratio) and a specific fragment after collision-induced dissociation is achieved for compounds where there is no isobaric similar compound. There may be the chance that some very unstable compounds might dissociate in the ion source and yield unspecific readings. However, this is highly variable across the different amino acids. Due to specific precursors and product ions created by the tandem mass spectrometers, this method achieves a sensitivity and specificity up to 99% and 99.9%, respectively, for most amino acid disorders, organic acidemias, and fatty acid oxidation defects [2, 13]. Liquid chromatography tandem mass spectrometry (LC-MS/MS) can analyze each sample quickly in 2 minutes, can allow multiple analytes to be tested using only a small amount of blood, and can provide high-volume throughput with rapid turnaround time. False positives are approximately 0.05%, which is less than the HPLC method. The accuracy is more than 90%, and the coefficient of variation (CV) is between 4 and 10% [14–16]. However, the accuracy and CVs are highly variably across the different types of amino acids [15]. Thus, a level of expertise is needed for preparing samples, operating the system, and interpreting the data. Despite the high initial capital cost for equipment, reagent cost is relatively low [2].

Reference values are crucial for interpreting test results and for making diagnoses [11, 17, 18]. Pediatric population requires the use of references that reflect rapid physiologic changes associated with growth but are often difficult to establish because of the challenges related to obtaining sufficient numbers of samples from healthy children [19]. There are no well-established reference intervals of amino acids in our population, and data from international literatures are traditionally used [20]. Levels of amino acids in the blood are influenced by several factors, such as age, sex, race, fasting time, nutritional status, illnesses, and medications [20–23]. A previous reference study in Thailand in 2001 was based on plasma amino acid analysis by the HPLC [24]. However, due to limited resources, the analysis method with plasma samples is not widely used in Thailand. Moreover, since the collected specimens must be transported to academic or university hospitals, the shipping of specimens is more conveniently achieved as dried blood spot (DBS) samples. In addition, the changing of environment and nutrients' composition over time, in particular protein intake, in young children might affect amino acid levels [22]. Reference values derived by different analysis methods cannot be used interchangeably, and data obtained at a given laboratory are recommended to provide quality services [25]. There are emerging researches involved with reference values of amino acids around the world [22, 26–29]. The aim of this study was to establish the age-related reference intervals for amino acids by LC-MS/MS, which had never been done in Thailand. Secondary objective was to assess factors that might affect amino acids.

## 2. Materials and Methods

### 2.1. Study Design

This cross-sectional descriptive study was conducted at the King Chulalongkorn Memorial Hospital (KCMH) from March 2016 to May 2017 with approval from the Institutional Review Board of the Faculty of Medicine, Chulalongkorn University, and informed consent was obtained from the parents. Study sites included outpatient department, well-baby clinic, full-term neonatal ward, and settings in accordance with ongoing pediatric departmental activities or research projects from the same locality (e.g., pediatric infectious unit). The recruitment of healthy subjects was also carried by the social media of Chula Kids' Club (together with immunization program) and school health checkup programs.

### 2.2. Study Population

A total of 277 healthy infants and children from 0 to 12 years of age were included in this study. They were divided into five age groups: <4 days old, 6–12 months, 1–3 years, 3–6 years, and 6–12 years. The age ranges were selected to represent the physiologic periods, such as newborn period and early and late childhood [24]. We excluded the subjects with known inborn errors of metabolism, disorders for which variations of amino acid levels have been described [17, 20, 26, 27], such as skin disease, neurological disease, gastrointestinal disease, liver disease, kidney disease, endocrine disease, cancer, infection, known nutritional abnormalities, or were taking any medication. All infants were full term, appropriate for gestational age, had normal birth weight, and were not born to diabetic mothers.

### 2.3. Data Collection

Data collection from the medical records and physical examination were documented into the case record forms with the details of age, sex, weight, height/length, fasting time before collecting blood samples, and 24 hr dietary recall.

### 2.4. Sample Collection

For <4 day-old infants, blood samples were collected from the heel stick onto the filter paper (Whatman 903) as dried blood spots (DBSs). For children aged at least 6 months, blood samples were collected as heparinized blood 1-2 ml and then pipetted 55 *µ*l of blood onto the filter paper. Then, the blood spots were left to dry for 4 hours on a horizontal, nonhumid and nonabsorbent surface at ambient temperature. The specimens were protected in dry plastic bags and were stored and frozen at −20°C until analysis within 1 month. After the DBS was collected, the remaining whole blood samples were then centrifuged, and the plasma were stored frozen at −80°C for future use.

### 2.5. Analysis of Amino Acids

Analysis was performed with Waters Acquity TQ-S, using MassLynx 4.1 and NeoLynx 4.1 (Waters, Milford, MA, USA). Preparation of MassChrom® Amino Acids and Acylcarnitines from Dried Blood (Chromsystems, Munich/Germany) was in accordance with the certified manufacturer's specifications [14], as follows. A 3.2 mm dried blood spot disk was punched out of the filter card into a 96-well microtiter plate. 200 *µ*l of the reconstituted internal standard was added. The plate was sealed with a protective sheet and agitated for 20 min at 37°C and 600 rpm. The supernatant was transferred into a new microtiter plate. 150 *µ*l of the internal standard for succinylacetone was added to the remaining blood spots. The 96-well plate was sealed with a protective sheet and agitated at 600 rpm for 30 min at 60°C. The supernatants were pooled from after the addition of the internal standard for succinylacetone with the first supernatant and evaporated at 60°C and 600 rpm to dryness. 60 *µ*l derivatisation reagent (butan-1-ol, n-butyl acetate, and hydrogen chloride) was added, the microtiter plate was sealed with a protective sheet, incubated 15 min at 60°C and 600 rpm, and evaporated at 60°C and 600 rpm to dryness. 100 *µ*l reconstitution buffer was added and agitated for 1 min at 600 rpm. 10 *µ*l was injected into the LC-MS/MS system by an autosampler. The operating conditions had 1.7-minute run time and mobile phase gradient with a flow rate of 20–600 *µ*l/min (Table 1).

### 2.6. Statistical Analysis

Data analysis was performed using Stata version 13.1 (Stata Corp., College Station, Texas). For descriptive analysis, the frequencies and percentages of categorical variables for the population characteristics were calculated, while median, interquartile ranges (IQR), the 2.5th, and 97.5th percentiles were calculated for continuous variables. The Kruskal–Wallis test was used to compare continuous variables between age groups. Levels of amino acids in *µ*mol/L were expressed as median since they were not normally distributed according to tests for normality (Kolmogorov–Smirnov and Shapiro–Wilk) and were skewed to the right. Thus, reference intervals were determined nonparametrically and correspond to the 2.5th–97.5th percentiles of the distribution. Quantile regression analysis was used to determine the factors associated with amino acids in both univariate and multivariate models. Multivariate models were developed by including covariates with *p* < 0.1 in univariate models. All *p* values reported are two-sided. Statistical significance was defined as *p* < 0.05.

## 3. Results

Table 2 shows the characteristics of subjects including demographic data and dietary intake, displayed as median and interquartile range (IQR) and anthropometry as mean (SD). Regarding gender, 51.3% of 277 subjects were male. All subjects were in good nutritional state by WHO *z*-score criteria and had normal protein intake according to their age. Of note, the energy and macronutrients intake, including caloric distribution, was in accordance with the 4th Thai National Health Examination Survey in 2008–2009 (NHES IV) [30], with a slightly higher energy intake of the 3–6 years age group. Fasting duration before collecting the blood samples was displayed in minutes. Children aged 6–12 years had an overnight fast for their health checkups. Regarding the <4 days old age group, their average gestational age was 38.5 weeks with 3058 g birth weight. Dietary data of infants aged <4 days were not demonstrated, as all the subjects of this group were newborns aged 48–72 hours (mean age 58.3 hours) who were predominantly breastfed and had their blood taken simultaneously with the Thai National Thyroid Screening Program.

### 3.1. Amino Acid Results

Thirteen amino acid levels were reported as the median for each of the five age groups: <4 days old, 6–12 months, 1–3 years, 3–6 years, and 6–12 years, as shown in Table 3. The age-specific reference intervals covered the central 95 percent of the test results, as represented by the 2.5th percentile and 97.5th percentile, respectively, according to the CLSI EP28-A3c guideline [31]. Median concentrations were significantly different for all amino acids when compared across age groups using the Kruskal–Wallis test (mostly *p*=0.0001). Since the levels of each amino acid showed no significant differences between males and females, the data were considered together. The isobaric analytes of leucine, isoleucine, alloisoleucine, and hydroxyproline could not be separated by this method. They were summed up under the same mass-to-charge ratio and reported as “leucine/isoleucine” concentrations in this study.

### 3.2. Trends of Age-Specific Concentration of Amino Acids

**Figure 1:**
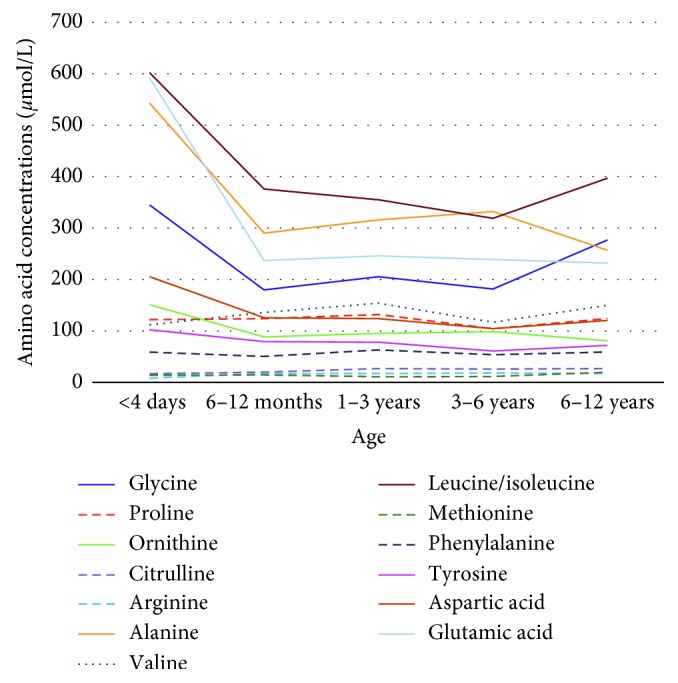
Trends of the age-specific distribution of each amino acid median concentration.

Graphic forms showing trends of the age-specific distribution of amino acid median concentrations are illustrated in Figure 1. Although each amino acid possessed its own unique pattern of distribution, we can identify some common tendencies into two major different profiles.

The first group consisted of seven amino acids (glycine, ornithine, alanine, leucine/isoleucine, tyrosine, aspartic acid, and glutamic acid) that drop sharply from the zero-time point collected shortly after birth to the median of the 6–12 month age group. Especially glycine, alanine, leucine/isoleucine, and glutamic acid demonstrated a sharp decrease in their concentrations approximately to half of their initial amounts. Afterwards, their concentrations remained stable; except for glycine and leucine/isoleucine which increased throughout older childhood (after 6 years of age) and alanine which peaked at 3–6 years and decreased afterwards.

The second group consisted of six others (citrulline, arginine, methionine, proline, phenylalanine, and valine) which showed steady concentrations throughout infancy and childhood. Citrulline, arginine, and methionine were at low levels (below 30 *µ*mol/L), while proline and phenylalanine were at middle levels of 50–130 *µ*mol/L and slightly increased during preadolescence. Lastly, the level of valine tended to slightly increase up to 3 years of age, followed by a period of modest decrement and subsequently rise again after 6 years of age.

### 3.3. Factors Affecting Amino Acids

Factors that might affect amino acids such as demographic data, fasting duration, and dietary intake were analyzed through quantile regression aimed at estimating the conditional median in both univariate and multivariate models. Initially, univariate analysis demonstrated several potential factors related to amino acid concentrations such as sex, energy intake, protein intake, and fasting time (data in Supplementary Table 1). Univariate analysis of fasting time showed significant association with alanine, glycine, methionine, and citrulline. However, multivariate models adjusting for these potential covariates with *p* < 0.1 in univariate analysis revealed that the remaining significant associated factor was only energy intake on glycine and phenylalanine. Subsequently, the acquired coefficient values for each amino acid were interpreted to demonstrate the effects. For every 1 Kcal/kg/day increased energy intake, glycine decreased by 0.7 *µ*mol/L (*p*=0.01) and phenylalanine decreased by 0.1 *µ*mol/L (*p*=0.03). After being adjusted in the multivariate models, protein intake and fasting time were not significant factors. In addition, we did not find any further associated factors through quantile regression analysis aimed at estimating the higher conditional 90th percentiles (data not shown).

### 3.4. Quality Control

The samples were run on multiple assay plates. Each sample was run with its own internal standard use as calibrators, and 2 controls with known concentrations of the analyte for a high and a low value were included on each plate for every 20 specimens. The accuracies were determined by comparing the test results of 2 controls of the analytes (a high and a low value) with the known target values (provided from the test kit). Also, the test values of these controls had to fall within the given suggested ranges from the instruction manual. Then, we calculated the percentage of the test values to the known target values for each result. Finally, the percentage ranges were listed for each analytes. The precisions were determined by the coefficient of variation (CV). The number (N) of values used to calculate the CVs is 14 each for high and low values.

The results of the intra-assay CVs as shown in Table 4 were satisfactory [32]. The variation found in the interassay of more than 10%, but not exceeding 15%, was observed for ornithine, alanine, valine, and aspartic acid and was acceptable [32] considering that methods with pipetting, such as the one used for amino acid analysis, usually yield CVs between 10% and 15% [20, 33]. The accuracy for each amino acid was acceptable, mostly at 85–105%, except for the underestimation of arginine and overestimation of valine at the low target. Comparing the results obtained with the previous study with LC-MS/MS [15], it was observed that all of the CVs and accuracies found in this study were equivalent or better.

## 4. Discussion

### 4.1. Study Population and Methodology

We studied a group of 277 Thai children from birth to 12 years of age, and the number of subjects included in this study yielded larger sample size than that in most studies of pediatric references at other sites including Asia [20, 24, 27, 28]. Previous data regarding amino acids in the Thai pediatric population were very limited. Our study defined reference intervals for whole blood amino acids in only healthy pediatric population and did not include children admitted to the hospital with minor illnesses, contrary to some previous studies [20, 24]. We believe that our subjects who were all recruited in the urban area of Bangkok can sufficiently represent the Thai pediatric population because geographical distribution does not directly affect amino acids, and our results of dietary intake, especially protein, were in accordance with the 4th Thai National Health Examination Survey (NHES IV) [30]. Although, an experimental study in Mexico showed that ingestion of an urban diet induced a higher increase in the plasma concentration of some amino acids than ingestion of a rural diet, the amount of protein consumed in the urban diet was greater than in the rural diet [34].

The recruitment of infants aged <4 days as the first age group was primarily intended for future implementation of newborn screening and could result in the selection bias. However, the cultural tradition that healthy infants under 6 months of age in Thailand rarely have their blood taken leads to the limitation of finding sufficient numbers of participants of this age group. This suggests the room for improvement in the future.

Analysis of amino acids in this study was performed by LC-MS/MS and demonstrated reliable precision and accuracy comparable with previous reports of LC-MS/MS in newborns [15, 19] and other analytical methods such as HPLC while maintaining the advantage of short analysis time. Fingerhut et al. found that the accuracy and CVs were highly variable across the different analytes, in a way that basic amino acids (CV 6.4–18.1%) and acidic amino acids (CV 7–13.7%) demonstrated higher CVs when compared with neutral amino acids (CV 3.3–5%) [15]. However, we could not demonstrate the differences.

### 4.2. Trends of Age-Specific Concentration of Amino Acids

The age-related median values of seven amino acids (glycine, ornithine, alanine, leucine/isoleucine, tyrosine, aspartic acid, and glutamic acid) drop sharply from the zero-time point collected shortly after birth to the median of the 6–12 month age group (Figure 1). Previous studies in Thailand have not been able to demonstrate the drop of amino acids during the age interval of 0–6 months [24]. It remains possible that the values drop sharply sooner than before 6 months of age if given another age group between these two. The gap of knowledge during the 0–6 months of age period should be further explored.

### 4.3. Comparison of Amino Acids with Previous Studies

Normal amino acid values in children vary considerably in different reports [24]. Because there is no standard age-specific reference of amino acids for LC-MS/MS in Thai children, our results were compared with those of the study of Lepage et al. in Caucasian children by ion-exchange chromatography [20]. Thai reference was different in that most amino acid concentrations were higher, except for the same ranges of citrulline, valine, proline, methionine, and lower arginine.

Most amino acids of Thai newborn in this study were comparable with the recent study of LC-MS/MS on DBSs conducted only in newborns aged 0–4 days in the USA [19]. However, some amino acid levels were still higher, notably ornithine, leucine/isoleucine, glutamic acid, and alanine. Because our method showed satisfactory accuracy with known analytes, the reasons for these discrepancies could result from the difference in the ethnicity but might be secondary to other preanalytical errors such as technical problems regarding blood sample collection and storage, matrix effects [35], internal interferences (asparagine on ornithine and hydroxyproline on leucine) [36], and differences in extraction efficiency. Although hemolysis was found to cause increases in ornithine, aspartic acid, glutamic acid, and decreased arginine [37], it should not affect whole blood samples as collected in this study.

Compared with the study using different analysis method of HPLC in Thai children [24], six amino acids (glycine, ornithine, alanine, leucine/isoleucine, aspartic acid and glutamic acid) were higher in our study. This means that we should not directly use the reference intervals from different analytical methods. The accuracy, intra-assay CVs, and interassay CVs of HPLC [28] and our study were approximately the same, which suggested that both analytical methods are acceptable. The primary explanations for these differences may lie within the method of specimen collection as dried blood spots (DBSs), which was primarily designed for newborn screening, versus plasma specimens. Even with cautious handling, small concentration gradients of analytes might be present during spreading and drying of blood on the filter paper; combined with the effect of hematocrit, this can cause an overall imprecision of approximately 10% [2]. Likewise, mildly elevated glutamate concentrations can result from the age of the specimens allowing for glutamine to glutamate conversion [23], even when stored at the recommended temperature. In order to overcome these problems, we propose more “transference studies” for comparison of plasma and DBS samples [2].

The discordance regarding common trends of amino acid pattern profiles was also observed. While arginine, methionine, proline, and phenylalanine demonstrated a decrease in their concentrations during the first year of life in Caucasian children, they differently showed steady concentrations in our study. Nevertheless, aspartic acid and glutamic acid similarly demonstrated decreasing values. Valine also showed the same trend throughout infancy and childhood. The discrepancy between the previous studies and our work could be due to differences in methodology, equipment, population, measurement conditions, or from dietary patterns which were not elucidated.

### 4.4. Factors Affecting Amino Acids

Age was confirmed to be an important factor because median concentrations for all amino acids across age groups were significantly different, similar to previous studies in Thailand and Spain [20, 24, 27]. This study showed no significant differences for amino acids between both genders, in contrast with previous studies in Turkey and China [26, 28].

From the quantile regression analysis (Supplementary Table 1), the only significant associated factor was energy intake on glycine and phenylalanine. For every 1 Kcal/kg/day increased energy intake, glycine decreased by 0.7 *µ*mol/L (*p*=0.01) and phenylalanine decreased by 0.1 *µ*mol/L (*p*=0.03). However, the changes in amino acid concentrations, acquired as coefficient values, were so small as to probably have limited clinical significance. Glycine is a nonessential amino acid and is a biomarker for diagnosis of nonketotic hyperglycinemia. While phenylalanine is an essential amino acid, the observed effect on the concentrations seemed to be too minute to have important clinical impact. Further researches specially designed to study the effect of these potential factors should be developed.

Protein intake was not a significant factor after being adjusted in the multivariate models from quantile regression analysis. This could result from inadequate power to detect minuscule differences between dietary protein intakes in the normal ranges of our subjects. Our study cannot identify the exact protein intake of newborns because we have no way of determining the protein content of the breast milk that the infant study participants consumed. However, previous data demonstrated that high dietary protein intake affected amino acids metabolism in infants aged 6 months [38] or may even be earlier at 4 months of age [22].

Univariate analysis of fasting time showed significant association with alanine, glycine, methionine, and citrulline. For example, every 1 minute of increased fasting duration, alanine decreased by 0.1 *µ*mol/L (*p*=0.001) which was the largest change of concentrations observed. This could be explained with gluconeogenesis from this glucogenic amino acid during the postabsorptive state. However, it was also an insignificant factor after being adjusted in the multivariate models. Moreover, the effect size on the change of amino acid concentrations seemed not to be large enough to have substantial clinical impact. Previous studies tended to fast children after 2 years of age for 8–10 hours [20, 26, 28] before blood collection, while some studies did not mention about the duration of fasting [24, 27]. However, no study clearly elucidated the influence of fasting on amino acids in human. Hence, we suggested that with this method, fasting should not always be obliged in children, especially in infants. This complies with current practices which do not require prolong fasting before blood collection in infants and younger children for convenience, along with reducing distress and chances of hypoglycemia.

Despite some outliers of amino acid levels, all subjects were confirmed to be doing well without any abnormal clinical status by tracing back from the case record forms and telephone contact. Also, there was no report from the subjects' parents to the investigators or nutrition unit staffs regarding the subjects' irregularity. These data were suggested to be resulted from *in vitro* or technical variations.

### 4.5. Clinical Applications

For the reason of the diagnosis of inborn errors of metabolism, the abnormal levels of amino acids should be significantly apparent. Although our subjects of age <4 days were newborns aged 48–72 hours who were predominantly breastfed, the patients with inborn errors of metabolism mostly have distinctly elevated metabolites and should be detected with this reference, especially when combined with suspected clinical presentations. Also, for the reason of biochemical follow-up, the trend of amino acid changes over time along with the patients' clinical profiles that could guide us for the management. Valine at the lower concentration tended to be overestimated. While this should not be problematic for the diagnosis of inborn errors of metabolism, it could result in underdetection of the deficiency during the monitoring period. From this study, amino acids such as phenylalanine, tyrosine, and methionine were reliable and within previous reported ranges [19, 24]. Hence, the benefit was obtained for diseases, such as phenylketonuria and tyrosinemia, on the diagnosis and monitoring. Nevertheless, this study may have less impact on the diseases such as maple syrup urine disease (MSUD) because leucine and isoleucine levels cannot be separated. When MSUD patients were on nutritional management with dietary restriction of BCAAs especially leucine, monitoring of each amino acid is important as to avoid excess and deficiency. Thus, without the complete information of all common amino acids, defects of clinical decision may occur. There were some occasions of leucine excess with isoleucine deficiency, and these scenarios cannot be detected by this method.

### 4.6. Considerations and Plans for Future Researches

To the best of our knowledge, this is the first study in Thai children population examining amino acids with LC-MS/MS. The strengths of our study include larger sample size compared with most studies of pediatric references, information on nutritional status, and dietary intake of the subjects. Analysis of amino acids by LC-MS/MS demonstrated reliable precision and accuracy while maintaining the advantage of short analysis time. The specimen collection from the heel stick onto the filter paper as dried blood spots is currently utilized as a newborn screening tool in other countries and Thailand [2] because it can be done with ease and requires less blood than plasma specimens. Moreover, the DBS samples can be conveniently transported to academic centers with laboratory facilities. We suggest that whole blood amino acids might be collected at <4 days age for the purpose of expanded neonatal screening simultaneously with the routine newborn screening in Thailand and fasting may not always be necessary. This study was also the first to expand the application of the analysis onto different pediatric age groups. Nevertheless, the gap of knowledge during the 0–6 months of age period should be further explored.

Major limitations include incomplete information of all common amino acids and inability to separate leucine from isoleucine and other isobaric analytes. There are rooms for improvement on the analytical methods and procedures for the laboratory. For instance, a method of LC-MS/MS compatible with the current Waters Acquity TQ-S machine at the central laboratory, KCMH, using AbsoluteIDQ® p180 test kit (Biocrates Life Sciences AG, Austria) is to be developed in the near future. This reliable test kit which covered 20 common amino acids was studied in many recent researches on targeted metabolites and amino acids [39–41] but has never been used elsewhere in Thailand due to limited access and the need for plasma samples. Furthermore, there are published methods to overcome the problem regarding the separation and quantification of BCAAs in patients with MSUD from the initial extraction of the dried blood samples with a sensitive and rapid second-tier UPLC-MS/MS method [42].

We aim to develop more expertise regarding specimen preparation, system operation, and data interpretation. We suggest further studies involving amino acids in patients with inborn errors of metabolism. Also, data of acylcarnitines and succinylacetone which can aid in the diagnosis of inborn errors of metabolism should also be studied.

In the absence of established values for amino acids in the Thai population measured by LC-MS/MS, the present reference ranges may be used for the diagnosis and management of patients, which may result in less expected turnaround time and better patient care. We hope that this will be a reference for subsequent studies about amino acids in Thai children with various conditions which affect amino acids in the future.

## 5. Conclusion

LC-MS/MS is a rapid and reliable method for the measurement of amino acids. With different analysis methods, reference intervals for LC-MS/MS in the Thai pediatric population diverge from previous studies. Thus, for the optimal clinical practice, age-specific reference intervals of amino acids should be designated for the particular population and analysis method. The reference intervals established in this study may guide us in the diagnosis and management of inherited metabolic disorders, as well as other diseases that affect amino acid metabolism.

## Figures and Tables

**Table 1:** Operating conditions with changing flow rate during the run time for the analysis (adapted from Instruction Manual for LC-MS/MS Analysis MassChrom [14]).

Time (min)	0	0.24	0.25	1.24	1.25	1.5	1.51
Flow (*µ*l/min)	200	200	20	20	600	200	200

**Table 2:** Characteristics of 277 subjects regarding demographic data, anthropometry, and dietary intake (BM = breast milk predominant; C = carbohydrate; P = protein; F = fat).

*n*	<4 days old	6–12 months	1–3 years	3–6 years	6–12 years
	85	43	49	50	50
*Demographic Data*					
Age (month)	0.08^∗^ (0.07–0.09)	9 (7–11)	23 (18–28)	44 (40–57)	120 (108–138)
Sex: male, *n* (%)	43 (51)	20 (47)	21 (43)	24 (48)	34 (68)
*Anthropometry, mean (SD)*					
W/A *z*-score	−0.5 (0.52)	−0.26 (1.13)	−0.17 (1.13)	−0.33 (1.44)	0.35 (0.47)
W/H *z*-score	−0.72 (0.78)	0.06 (0.95)	0.11 (1.2)	0.13 (1.37)	−0.19 (0.75)
BMI *z*-score	−0.73 (0.63)	−0.02 (1)	0.21 (1.2)	0.08 (1.5)	0.2 (1.17)
*Dietary data*					
Energy (Kcal/day)	BM	973 (708–1121)	1049 (854–1402)	1304 (1144–1489)	1230 (1181–1382)
Energy (Kcal/kg/day)	BM	107 (86–130)	98 (80–124)	88 (74–100)	41 (33–48)
Protein (g/day)	BM	24 (15–32)	40 (35–52)	49 (42–56)	50 (40–65)
Protein (g/kg/day)	BM	3 (1.8–3.8)	3.9 (2.8–5.2)	3.2 (2.5–4)	1.7 (1.3–2.2)
C : P : F	BM	50 : 13 : 37	50 : 16 : 34	54 : 16 : 30	50 : 17 : 33
*Fasting time (min)*	—	120 (30–165)	135 (90–210)	140 (90–190)	≥720

^∗^Age: <4 days old; mean age: 58.3 hours. Data were expressed as median (IQR) unless stated otherwise.

**Table 3:** Distribution of amino acid concentrations by age groups, displayed as median, 2.5th, and 97.5th percentiles.

Amino acid (*µ*mol/L)	<4 days			6–12 months			1–3 years			3–6 years			6–12 years		
	Median	P 2.5	P 97.5	Median	P 2.5	P 97.5	Median	P 2.5	P 97.5	Median	P 2.5	P 97.5	Median	P 2.5	P 97.5
Glycine	345	300	414	179.9	146.3	251.6	205.5	168.2	332.2	181.6	157.1	229	276.9	257.1	305.9
Proline	122	97	150	124	101.2	155.1	132	107	183.4	104.5	88.9	143.7	124.3	111.6	155.1
Ornithine	151	124	185	88.5	69.3	122.5	95.3	70.2	137.1	98.7	82.4	111.3	80.9	68.7	88.4
Citrulline	16.9	13.8	23	20	18.5	25.6	27	21.9	31.8	26	22.1	31.8	27	24.4	28.8
Arginine	8.4	5.6	14.7	17.2	14.2	23.5	17.4	12.7	25.1	18.4	13.8	25.8	17.2	15.4	20.7
Alanine	543	424	633	290.3	253.5	355.8	316	258.1	406.2	332.2	293	386.9	256.9	221.6	307.8
Valine	112	89.1	179.2	136	118	160.3	154	120.1	205.3	117	103	174.4	150	128.4	171.4
Leucine/isoleucine	602	511	703	376	305.4	440.1	355	282.9	436.2	319	275.3	396.1	397	343	472.5
Methionine	14.1	10.7	17.7	14.6	9.4	21.9	11.1	7	18.5	11.5	8.2	15.6	19.8	17.1	22.6
Phenylalanine	59	50.3	70.1	50.5	40.5	62.7	63.2	49.3	72.3	53.9	47.1	84.2	59.2	53.3	66.2
Tyrosine	91.1	74.1	114.4	73.5	52.6	85.5	78	58.3	93.3	61.1	50.1	91.3	72.1	62.3	84.3
Aspartic acid	205.6	102	216	125.7	94	157.4	124	87.9	212.4	104.5	85.7	137.8	120.5	88.1	148.8
Glutamic acid	592	485	687.4	237	194	306	246	201.2	283.8	239	214.8	262.3	232	211	258.7

The age-specific reference intervals were represented by the 2.5th and 97.5th percentiles according to the CLSI EP28-A3c guideline [31].

**Table 4:** Accuracy, intra-assay, and interassay CV (%) of each amino acid at two different concentrations.

Analyte (*µ*mol/L)	Low target	Accuracy (%)	Intra-assay CV (%)	Interassay CV (%)	High target	Accuracy (%)	Intra-assay CV (%)	Interassay CV (%)
Glycine	355	98–100	5.7	4.8	1018.5	99.5–101.6	5.7	8.3
Proline	271.5	100–101	2.8	4.5	695	62.5–100	2.1	2.5
Ornithine	219	64.9–82.4	2.5	13.1	519	65.8–85.6	1.2	11.7
Citrulline	86	88.2–94.5	2.1	9.4	313	79.5–93	2.4	7.2
Arginine	113	47.2–56.2	1.3	2.3	240	61.3–63.7	1.6	2.9
Alanine	320	100–110.2	2.5	9	563	108–138.3	7.5	11.2
Valine	159	124.5–128.4	7.1	8.2	361	85.5–106.4	9	12.2
Leucine/isoleucine	224	110.3–137	5.5	7.6	537	97.7–115.4	4	4.5
Methionine	46	99.3–100	5.2	8.6	172	100	2.3	4.5
Phenylalanine	97	106.4–115.4	2.8	5.8	420	91.3–101.8	0.7	2
Tyrosine	175	99.8–105	3.1	3.8	536	83.4–95.2	1.6	2.9
Aspartic acid	161	102.1–106.6	2	10.5	282	107.5–112.3	6.8	7.1
Glutamic acid	489	91.8–93.4	3.6	7.4	743	77.1–90.3	2.7	5.3

## Supplementary Materials

Supplementary Materials Supplementary Table 1: factors affecting blood amino acids in both univariate and multivariate models. Description: factors that might affect blood amino acids such as age, sex, fasting duration, and dietary intake were analyzed through quantile regression aimed at estimating the conditional median in both univariate and multivariate models. Initially, univariate analysis demonstrated several potential covariates with *p* < 0.1. However, the multivariate models revealed that the remaining significant associated factor was only energy intake on glycine and phenylalanine (*p* < 0.05). The acquired coefficient values for each amino acid were then interpreted to demonstrate the effect size.
