# Supplementary material for: Age-Related Reference Intervals for Blood Amino Acids in Thai Pediatric Population Measured by Liquid Chromatography Tandem Mass Spectrometry
Source: J Nutr Metab. 2018 May 6;2018:5124035. doi: 10.1155/2018/5124035 (PMC5960525; doi:10.1155/2018/5124035)
Supplement: Supplementary Materials — Supplementary Table 1: factors affecting blood amino acids in both univariate and multivariate models. Description: factors that might affect blood amino acids such as age, sex, fasting duration, and dietary intake were analyzed through quantile regression aimed at estimating the conditional median in both univariate and multivariate models. Initially, univariate analysis demonstrated several potential covariates with p < 0.1. However, the multivariate models revealed that the remaining significant associated factor was only energy intake on glycine and phenylalanine (p < 0.05). The acquired coefficient values for each amino acid were then interpreted to demonstrate the effect size. [file 5124035.f1.docx]

**Supplement Table 1** Factors affecting blood amino acids in both univariate and multivariate models

|  | **Univariate** | | | **Multivariate** | | |  |
| --- | --- | --- | --- | --- | --- | --- | --- |
|  | **Coefficient** | **95%CI** | **P** | **Coefficient** | **95%CI** | **P** |  |
| **Glycine** |  |  |  |  |  |  |  |
| Age (months)  0-6 month |  |  | 0.01 |  |  | 0.83 |  |
| 6-12 mo | ref | - |  | ref | - |  |  |
| 1-3 yr | 25.6 | -14.2 to 65.3 |  | 35.9 | -4.5 to 76.4 |  |  |
| 3-6 yr | 3.1 | -36.5 to 42.6 |  | -12.3 | -53.2 to 28.6 |  |  |
| 6-12 yr | 97.0 | 57.4 to 136.5 |  | 86.6 | -13.9 to 187.1 |  |  |
| Male | 48.4 | 12 to 84.8 | 0.009 | -2.5 | -30.4 to 25.4 | 0.86 |  |
| Fasting (min) | 0.15 | 0.1 to 0.21 | <0.001 | 0.01 | -0.2 to 0.1 | 0.63 |  |
| **Energy** | **-1.04** | **-1.4 to -0.6** | **<0.001** | **-0.7** | **-1.3 to -0.2** | **0.01** |  |
| Protein | -8.9 | -17.8 to 0.07 | 0.05 | 10.2 | -2.2 to 18.3 | 0.15 |  |
| **Proline** |  |  |  |  |  |  |  |
| Age (months)  0-6 month |  |  | 0.80 |  |  |  |  |
| 6-12 mo | ref | - |  |  |  |  |  |
| 1-3 yr | 8.0 | -16.3 to 32.3 |  |  |  |  |  |
| 3-6 yr | -19.0 | -43.1 to 5.1 |  |  |  |  |  |
| 6-12 yr | 0.4 | -23.8 to 24.5 |  |  |  |  |  |
| Male | -2.1 | -17.8 to 13.7 | 0.80 |  |  |  |  |
| Fasting (min) | 0.0 | 0 to 0 | 0.75 |  |  |  |  |
| Energy | -0.1 | -0.4 to 0.1 | 0.19 |  |  |  |  |
| Protein | 2.3 | -1.4 to 5.9 | 0.22 |  |  |  |  |
| **Ornithine** |  |  |  |  |  |  |  |
| Age (months)  0-6 month |  |  | 0.24 |  |  |  |  |
| 6-12 mo | ref | - |  |  |  |  |  |
| 1-3 yr | 6.8 | -10 to 23.6 |  |  |  |  |  |
| 3-6 yr | 10.3 | -6.4 to 27 |  |  |  |  |  |
| 6-12 yr | -8.6 | -25.3 to 8.1 |  |  |  |  |  |
| Male | 1.3 | -7.6 to 10.2 | 0.78 |  |  |  |  |
| Fasting (min) | 0.0 | 0 to 0 | 0.07 |  |  |  |  |
| Energy | 0.1 | -0.1 to 0.2 | 0.21 |  |  |  |  |
| Protein | 1.8 | -0.4 to 4 | 0.11 |  |  |  |  |
| **Citrulline** |  |  |  |  |  |  |  |
| **Age (months)**  **0-6 month** |  |  | **<0.001** |  |  | **0.01** |  |
| **6-12 mo** | **ref** | **-** |  |  |  |  |  |
| **1-3 yr** | **7.0** | **4.3 to 9.7** |  | **5.4** | **2.4 to 8.4** |  |  |
| **3-6 yr** | **6.0** | **3.3 to 8.7** |  | **4.5** | **1.5 to 7.5** |  |  |
| **6-12 yr** | **7.1** | **4.4 to 9.8** |  | **-0.4** | **-7.7 to 6.9** |  |  |
| Male | -0.6 | -2.8 to 1.6 | 0.59 |  |  |  |  |
| Fasting (min) | 0.01 | 0.001 to 0.01 | 0.01 | 0.01 | -0.0001 to 0.02 | 0.05 |  |
| Energy | -0.02 | -0.04 to 0.1 | 0.26 |  |  |  |  |
| Protein | 0.1 | -0.4 to 0.7 | 0.60 |  |  |  |  |
|  | **Univariate** | | | **Multivariate** | | |  |
|  | **Coefficient** | **95%CI** | **P** | **Coefficient** | **95%CI** | **P** |  |
| **Arginine** |  |  |  |  |  |  |  |
| Age (months)  0-6 month |  |  |  |  |  |  |  |
| 6-12 mo | ref | - | 0.93 |  |  |  |  |
| 1-3 yr | 0.2 | -3.9 to 4.3 |  |  |  |  |  |
| 3-6 yr | 1.3 | -2.7 to 5.3 |  |  |  |  |  |
| 6-12 yr | 0.1 | -3.9 to 4.1 |  |  |  |  |  |
| Male | 3.7 | 1.2 to 6.2 | 0.001 |  |  |  |  |
| Fasting (min) | -0.0001 | -0.005 to 0.005 | 0.94 |  |  |  |  |
| Energy | 0.004 | -0.029 to 0.036 | 0.82 |  |  |  |  |
| Protein | -0.1 | -0.7 to 0.5 | 0.84 |  |  |  |  |
| **Alanine** |  |  |  |  |  |  |  |
| Age (months)  0-6 month |  |  | 0.23 |  |  |  |  |
| 6-12 mo | ref | - |  |  |  |  |  |
| 1-3 yr | 25.7 | -15.4 to 66.9 |  |  |  |  |  |
| 3-6 yr | 45.7 | 4.8 to 86.7 |  |  |  |  |  |
| 6-12 yr | -33.1 | -74 to 7.9 |  |  |  |  |  |
| Male | -5.0 | -35.7 to 25.7 | 0.75 |  |  |  |  |
| Fasting (min) | -0.1 | -0.139 to -0.03 | 0.001 |  |  |  |  |
| Energy | 0.2 | -0.235 to 0.631 | 0.37 |  |  |  |  |
| Protein | -0.1 | -7.8 to 7.6 | 0.98 |  |  |  |  |
| **Valine** |  |  |  |  |  |  |  |
| Age (months)  0-6 month |  |  |  |  |  |  |  |
| 6-12 mo | ref | - | 0.38 |  |  |  |  |
| 1-3 yr | 18.0 | -5.5 to 41.5 |  |  |  |  |  |
| 3-6 yr | -19.0 | -42.4 to 4.4 |  |  |  |  |  |
| 6-12 yr | 13.0 | -10.4 to 36.4 |  |  |  |  |  |
| Male | 3.0 | -12.4 to 18.4 | 0.70 |  |  |  |  |
| Fasting (min) | 0.02 | -0.007 to 0.055 | 0.12 |  |  |  |  |
| Energy | -0.1 | -0.366 to 0.07 | 0.18 |  |  |  |  |
| Protein | 0.2 | -3.6 to 4.1 | 0.91 |  |  |  |  |
| **Leucine/ isoleucine** | |  |  |  |  |  |  |
| Age (months)  0-6 month |  |  |  |  |  |  |  |
| 6-12 mo | ref | - | 0.30 |  |  |  |  |
| 1-3 yr | -21.0 | -76.5 to 34.5 |  |  |  |  |  |
| 3-6 yr | -56.0 | -111.3 to -0.7 |  |  |  |  |  |
| 6-12 yr | 22.0 | -33.3 to 77.3 |  |  |  |  |  |
| Male | 12.0 | -24 to 48 | 0.51 |  |  |  |  |
| Fasting (min) | 0.1 | -0.013 to 0.121 | 0.12 |  |  |  |  |
| Energy | -0.3 | -0.789 to 0.182 | 0.22 |  |  |  |  |
| Protein | -0.7 | -9.2 to 7.9 | 0.88 |  |  |  |  |
|  | **Univariate** | | | **Multivariate** | | |  |
|  | **Coefficient** | **95%CI** | **P** | **Coefficient** | **95%CI** | **P** |  |
| **Methionine** |  |  |  |  |  |  |  |
| Age (months)  0-6 month |  |  |  |  |  |  |  |
| 6-12 mo | ref | - | 0.002 |  |  | 0.25 |  |
| 1-3 yr | -3.6 | -7.5 to 0.3 |  | -3.3 | -7.2 to 0.6 |  |  |
| 3-6 yr | -3.0 | -6.9 to 0.8 |  | -2.6 | -6.6 to 1.3 |  |  |
| 6-12 yr | 5.2 | 1.3 to 9 |  | 4.8 | -4.9 to 14.5 |  |  |
| Male | -0.1 | -3.2 to 3 | 0.95 |  |  |  |  |
| Fasting (min) | 0.01 | 0.008 to 0.017 | 0.001 | 0.002 | -0.012 to 0.016 | 0.79 |  |
| Energy | -0.1 | -0.103 to -0.028 | 0.001 | 0.007 | -0.04 to 0.1 | 0.78 |  |
| Protein | -0.4 | -1.2 to 0.3 | 0.25 |  |  |  |  |
| **Phenylalanine** |  |  |  |  |  |  |  |
| Age (months)  0-6 month |  |  |  |  |  |  |  |
| 6-12 mo | ref | - | 0.12 |  |  |  |  |
| 1-3 yr | 12.7 | 3.8 to 21.6 |  |  |  |  |  |
| 3-6 yr | 3.5 | -5.3 to 12.3 |  |  |  |  |  |
| 6-12 yr | 8.9 | 0.1 to 17.7 |  |  |  |  |  |
| Male | 1.6 | -3.6 to 6.8 | 0.55 |  |  |  |  |
| Fasting (min) | 0.01 | -0.001 to 0.018 | 0.08 | 0.005 | -0.02 to 0.01 | 0.53 |  |
| **Energy** | **-0.1** | **-0.2 to -0.02** | **0.02** | **-0.1** | **-0.2 to -0.02** | **0.03** |  |
| Protein | 0.5 | -0.8 to 1.7 | 0.47 |  |  |  |  |
| **Tyrosine** |  |  |  |  |  |  |  |
| Age (months)  0-6 month |  |  |  |  |  |  |  |
| 6-12 mo | ref | - | 0.49 |  |  |  |  |
| 1-3 yr | 4.5 | -8.6 to 17.6 |  |  |  |  |  |
| 3-6 yr | -11.3 | -24.4 to 1.8 |  |  |  |  |  |
| 6-12 yr | -1.0 | -14.1 to 12.1 |  |  |  |  |  |
| Male | -1.4 | -10.1 to 7.3 | 0.75 |  |  |  |  |
| Fasting (min) | 0.01 | -0.012 to 0.022 | 0.56 |  |  |  |  |
| Energy | -0.1 | -0.172 to 0.057 | 0.32 |  |  |  |  |
| Protein | 1.8 | -0.3 to 3.9 | 0.09 |  |  |  |  |
| **Aspartic acid** |  |  |  |  |  |  |  |
| Age (months)  0-6 month |  |  |  |  |  |  |  |
| 6-12 mo | ref | - | 0.08 | ref | - | 0.77 |  |
| 1-3 yr | 32.5 | 3.7 to 61.3 |  | 20.3 | -11.1 to 51.6 |  |  |
| 3-6 yr | 13.5 | -15.1 to 42.1 |  | 2.3 | -29.4 to 34 |  |  |
| 6-12 yr | 29.5 | 0.9 to 58.1 |  | -49.2 | -127.5 to 29.1 |  |  |
| Male | 7.0 | -12.1 to 26.1 | 0.47 |  |  |  |  |
| Fasting (min) | 0.01 | 0.001 to 0.074 | 0.04 | 0.2 | -0.05 to 0.1 | 0.53 |  |
| Energy | -0.2 | -0.497 to 0.005 | 0.06 | -0.1 | -0.5 to 0.3 | 0.78 |  |
| Protein | -1.0 | -5.4 to 3.4 | 0.65 |  |  |  |  |

|  | **Univariate** | | | **Multivariate** | | |
| --- | --- | --- | --- | --- | --- | --- |
|  | **Coefficient** | **95%CI** | **P** | **Coefficient** | **95%CI** | **P** |
| **Glutamic acid** |  |  |  |  |  |  |
| Age (months)  0-6 month |  |  |  |  |  |  |
| 6-12 mo | ref | - | 0.51 |  |  |  |
| 1-3 yr | 9.0 | -20.5 to 38.5 |  |  |  |  |
| 3-6 yr | 1.0 | -28.3 to 30.3 |  |  |  |  |
| 6-12 yr | -5.0 | -34.3 to 24.3 |  |  |  |  |
| Male | -8.0 | -26 to 10 | 0.38 |  |  |  |
| Fasting (min) | 0.01 | -0.042 to 0.025 | 0.63 |  |  |  |
| Energy | -0.1 | -0.369 to 0.132 | 0.35 |  |  |  |
| Protein | 1.5 | -3.2 to 6.1 | 0.54 |  |  |  |

Description:

Factors that might affect blood amino acids such as age, sex, fasting duration and dietary intake were analyzed through quantile regression aimed at estimating the conditional median in both univariate and multivariate models. Initially, univariate analysis demonstrated several potential covariates with p<0.1. However, the multivariate models revealed that the remaining significant associated factor was only energy intake on glycine and phenylalanine (p<0.05). The acquired coefficient values for each amino acid were then interpreted to demonstrate the effect size.
